# p53 Is a Key Regulator for Osthole-Triggered Cancer Pathogenesis

**DOI:** 10.1155/2014/175247

**Published:** 2014-06-12

**Authors:** Ssu-Ming Huang, Cheng-Fang Tsai, Dar-Ren Chen, Min-Ying Wang, Wei-Lan Yeh

**Affiliations:** ^1^Graduate Institute of Biotechnology, National Chung Hsing University, Taichung 402, Taiwan; ^2^Preventive Medicine Center, Department of Community Medicine, Taichung Tzu Chi Hospital, Buddhist Tzu Chi Medical Foundation, Taichung 427, Taiwan; ^3^Division of Colon and Rectal Surgery, Department of Surgery, Taichung Tzu Chi Hospital, Buddhist Tzu Chi Medical Foundation, Taichung 427, Taiwan; ^4^School of Medicine, Tzu Chi University, Hualien 970, Taiwan; ^5^Department of Biotechnology, Asia University, Taichung 413, Taiwan; ^6^Comprehensive Breast Cancer Center, Changhua Christian Hospital, Changhua 500, Taiwan; ^7^Department of Cell and Tissue Engineering, Changhua Christian Hospital, Changhua 500, Taiwan

## Abstract

Osthole has been reported to have antitumor activities via the induction of apoptosis and inhibition of cancer cell growth and metastasis. However, the detailed molecular mechanisms underlying the anticancer effects of osthole in human colon cancer remain unclear. In the present study, we have assessed osthole-induced cell death in two different human colon cancer cell lines, HCT116 and SW480. Our results also showed that osthole activated proapoptotic signaling pathways in human colon cancer cells. By using cell culture insert system, osthole reduced cell motility in both human colon cancer cell lines. This study also provides evidence supporting the potential of osthole in p53 activation. Expression of p53, an apoptotic protein, was remarkably upregulated in cells treated with osthole. Importantly, the levels of phosphorylation of p53 on Ser15 (p-p53) and acetylation of p53 on Lys^379^ (acetyl-p53) were increased under osthole treatment. Our results also demonstrated that p53 was activated followed by generation of reactive oxygen species (ROS) and activation of c-Jun N-terminal kinase (JNK). Our study provides novel insights of p53-mediated responses under osthole treatment. Taken together, we concluded that osthole induces cancer cell death and inhibits migratory activity in a controlled manner and is a promising candidate for antitumor drug development.

## 1. Introduction

Colon cancer is one of the leading causes of cancer-related deaths worldwide. Surgery can be applied in the early stage, while chemotherapy and/or radiation therapy are used to treat malignant tumors. The metastatic dissemination of primary tumors is linked directly to patients' survival and accounts for about 90% of all colon cancer deaths. It should therefore be obvious that tools and methodologies that allow early cancer detection will affect the survival time and rate of patients. The development of colon cancer is increasing in recent years; however, the knowledge of treatments is still limited.

The p53 tumor suppressor gene is one of the most commonly mutated genes found in human malignancies [1]. More than 50% of human cancer cells are associated with missense mutations or deletions of p53 [2], which results in chemoresistance of those cancer cells [3]. p53 protein is important in the transcription of growth inhibiting genes, apoptosis, cell cycle arrest, and DNA repair [4]. p53 is also a sequence-specific transcription factor that transactivates p21, which is involved in cell growth regulation [5]. The MAP kinase family comprises extracellular-signal-regulated kinase (ERK), p38, and c-Jun N-terminal kinase (JNK), and these signal pathways have been implicated in many physiologic processes, including cell growth and death through p53-dependent or p53-independent mechanisms [6]. Importantly, JNK signal transduction pathway is also activated by some anticancer drugs [7, 8]. JNK1 protein kinase is the major isoform in JNK family proteins that its activation requires phosphorylation on Thr^183^ and Tyr^185^ [9]. Dominant-negative mutation of JNK (DN-JNK) is a mutant at these sites (on Thr^183^ and Tyr^185^), which effectively suppresses paclitaxel-induced cell apoptosis in cancer cells [10].

Frequent consumption of natural fruits and vegetables has been considered to reduce risk of developing cancers and mortality [11, 12]. Osthole (7-methoxy-8-(3-methyl-2-butenyl)coumarin) is an active constituent isolated from* Cnidium monnieri* (L.)* Cusson,* which has been shown to exert a wide variety of biological effects such as contractility-based motility of different cells and tissues [13]. Osthole has also been shown to have anti-inflammatory [14], antiosteoporosis [15], and antiseizure [16] effects. In recent years, accumulating evidence also suggests that osthole has antitumor activities that are thought to occur via the induction of apoptosis and inhibition of cancer cell growth and metastasis [17–19].

ROS plays a key role in regulation of biological functions including differentiation and immune responses [20]. ROS is generated through a number of environmental stimuli, and excessive production of ROS causes oxidative stress leading to adverse events like cell death [21, 22]. Previous reports showed that the protective effects of osthole were revealed by reducing ROS production in ischemia-reperfusion injury models [23, 24]. However, the effect of osthole in generation of ROS in cancer cells is still unknown, and the detailed mechanisms underlying the anticancer effects of osthole in human colon cancer remain unclear. This study investigates the effects and underlying mechanisms of osthole-induced cell death and migration in human colon cancer. Our study reports that osthole induces cell death and reduces cell migration through the induction of ROS production, JNK activation, and p53 activation.

## 2. Experimental Section 

### 2.1. Reagents and Antibodies

Osthole was purchased from Sigma-Aldrich (St. Louis, MO). Fetal bovine serum (FBS), Dulbecco's modified Eagle's medium (DMEM), OPTI-MEM, and Lipofectamine 2000 (LF2000) were purchased from Gibco BRL (Invitrogen Life Technologies, Carlsbad, CA, USA). Primary antibodies against Bax, Bcl2, procaspase-3, PARP, JNK, and *β*-actin were purchased from Santa Cruz Biotechnology (Santa Cruz, CA). Primary antibodies against p-JNK Thr^183^/Tyr^185^, p-p53 Ser^15^, and acetyl-p53 Lys^379^ were purchased from Cell Signaling and Neuroscience (Danvers, MA). The primary antibody against p53 was purchased from BD Pharmingen Transduction Laboratories (San Diego, CA). The dominant-negative (DN) mutant of JNK plasmid was a gift from Dr. W.-M. Fu (National Taiwan University, Taipei, Taiwan).

### 2.2. Cell Culture

Human colon carcinoma cell lines HCT116 and SW480 were obtained from the American Type Culture Collection (Manassas, VA, USA). The procedures of cell culturing were conducted according to previous reports [25, 26].

### 2.3. Reactive Oxygen Species (ROS) Assay

The procedures of ROS assay were conducted according to previous reports [27, 28]. ROS production was determined by the oxidation of specific probe, H_2_DCFDA (2′,7′-dichlorodihydrofluorescein diacetate), by using flow cytometry. Cells were incubated with H_2_DCFDA (10 *μ*M) at 37°C for 30 min and then stimulated with osthole or hydrogen peroxide. Fluorescence intensities were determined with an excitation filter of 488 and 525 nm emission wavelengths.

### 2.4. Western Blot Analysis

Cells were lysed in a homogenization buffer on ice, and equal protein amounts of samples were loaded in an SDS-PAGE (polyacrylamide gel electrophoresis) [29]. The membrane was blocked with 5% nonfat milk and then probed with primary antibody. After several PBST washes, the membrane was incubated with a peroxidase-conjugated secondary antibody. The blots were visualized by enhanced chemiluminescence using Fuji medical X-ray film (Fujifilm, Tokyo, Japan). The blots were then stripped by incubation in stripping buffer [25] and reprobed a loading control. Quantitative data were obtained using a densitometer and Image J software (National Institute of Health, Bethesda, MA).

### 2.5. Migratory Activity Assay

*In vitro* migration assay was performed using Costar Transwell inserts (pore size, 8 *μ*m) (Corning, Albany, NY) as described previously [30–32]. Cells in 200 *μ*L of media were seeded in the upper chamber, and 300 *μ*L of media was placed in the lower chamber (incubated at 37°C in 5% CO_2_). After seeding cells in the upper chamber, cells were treated with osthole for 2 h. After a 24 h migration period, cells were stained with 0.05% crystal violet in 2% methanol. Nonmigratory cells on the upper surface of the filters were removed by wiping with a cotton swab. Cell number was counted in five random fields per well under a microscope at 200x magnification. Images of migratory cells were acquired using a digital camera and light microscope.

### 2.6. Reverse Transcriptase-PCR (RT-PCR)

Total RNA was extracted from cells using TRIzol kit (MDBio Inc., Taipei, Taiwan). The reverse transcription reaction was performed using 2 *μ*g of total RNA, which was reverse transcribed into cDNA using the oligo(dT) primer and then amplified using oligonucleotide primers:

p53: 5′-AATTTGCGTGTGGAGTATTT-3′ and

5′-GTGGAGTCTTCCAGTGTGAT-3′;

p21: 5′-AGGCACCGAGGCACTCAGAG-3′ and

5′-AGTGA CAGGTCCACATGGTCTTCC-3′;

GAPDH: 5′-TGGGCTACACTGAGCACCAG-3′ and

5′-GGGTGTCGCTGTTGAAGTCA-3′.

Each PCR cycle was carried out for 30 sec at 95°C, 30 sec at 55°C, and 1 min at 72°C. PCR products were then separated electrophoretically in a 2% agarose gel and stained with Novel Juice (GeneDireX, Las Vegas, Nevada).

### 2.7. MTT and SRB (Sulforhodamine B) Assays

The procedures of MTT and SRB assays determining cell viability were conducted according to previous reports [33–35]. After treatment with various concentrations of osthole for 24 h, cell culture media were aspirated. In MTT assay, MTT (0.5 mg/mL) was added to each culture well and incubated for 2 h at 37°C. The MTT reagent was then removed and washed with PBS for several times. DMSO (200 *μ*L per well) was added to dissolve formazan crystals. In SRB assay, cells were fixed* in situ* by gentle addition of 50 *μ*L per well of 10% TCA, and culture plates were incubated for 1 h at 4°C. After discarding supernatant, cells were then washed with PBS and 50 *μ*L of SRB solution was then added to each well for 10 min at room temperature. After staining, cells were washed with 1% acetic acid and dissolved in tris-base. The absorbance was determined at 550 nm (for MTT) or 515 nm (for SRB) using a microplate reader (Thermo Scientific, Vantaa, Finland).

### 2.8. Statistical Analyses

The values are reported as mean ± S.E.M. Statistical analyses between two groups were performed using Student's* t-*test. The difference was determined to be significant if the *P* value was <0.05.

## 3. Results 

### 3.1. Osthole Induces Cell Death and Apoptosis in Human Colon Cancer

**Figure 1:**
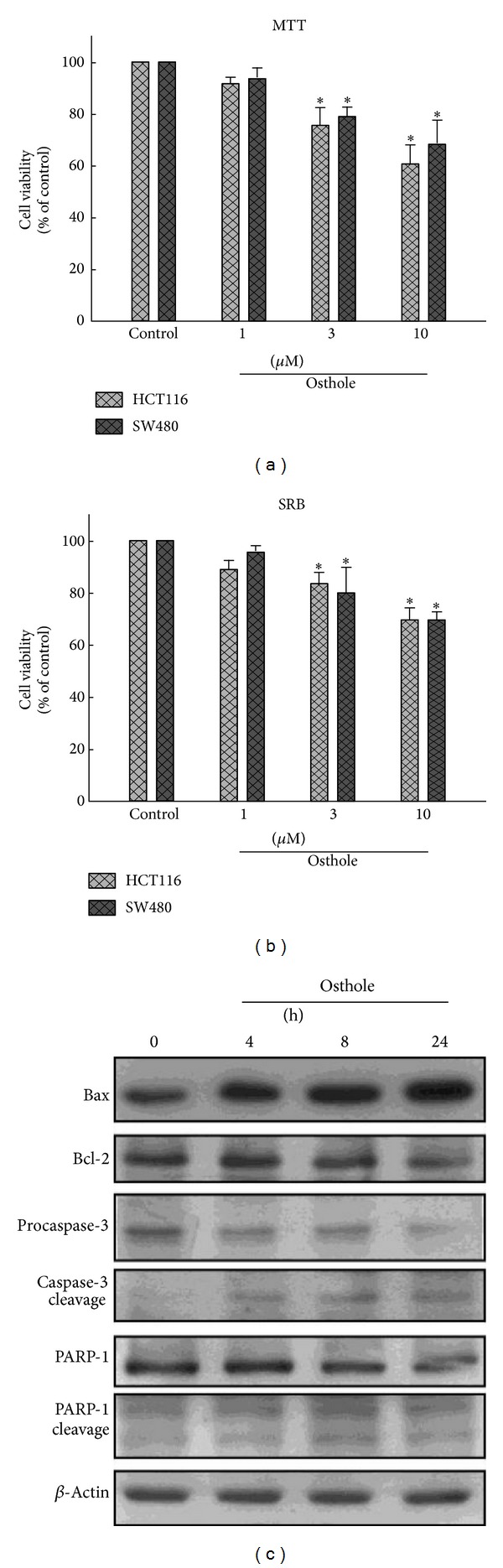
Osthole induces cell death in human colon cancer. Osthole-induced cell death of HCT116 and SW480 human colon cancer cells was shown. Cells were incubated with various concentrations of osthole (1, 3, or 10 *μ*M) for 24 h, and the cell viability was examined by MTT (a) or SRB (b) assays. Results are expressed as the means ± S.E.M. from three independent experiments. *P < 0.05, compared with the vehicle treatment group. (c) SW480 cells were incubated with osthole (10 *μ*M) for indicated time periods, and the protein expressions of Bax, Bcl-2, procaspase-3, cleaved caspase-3, and PARP-1 were examined by western blot analysis. Results are the representative of three independent experiments.

In order to investigate whether osthole affects cell viability, HCT116 and SW480 human colon cancer cells were incubated with various concentrations of osthole (1, 10, or 30 *μ*M) for 24 h, and cell viability of both cell lines was determined by MTT and SRB assay. We observed that osthole induced cell death of human colon cancer in a concentration-dependent manner (Figures 1(a) and 1(b)). Treatment of SW480 cells with osthole also increased Bax protein expression and the cleavage of caspase-3 and PARP-1. Meanwhile, osthole decreased the protein expression of Bcl-2, procaspase-3, and PARP-1 as well (Figure 1(c)).

### 3.2. Osthole Inhibits Migratory Activity of Human Colon Cancer

**Figure 2:**
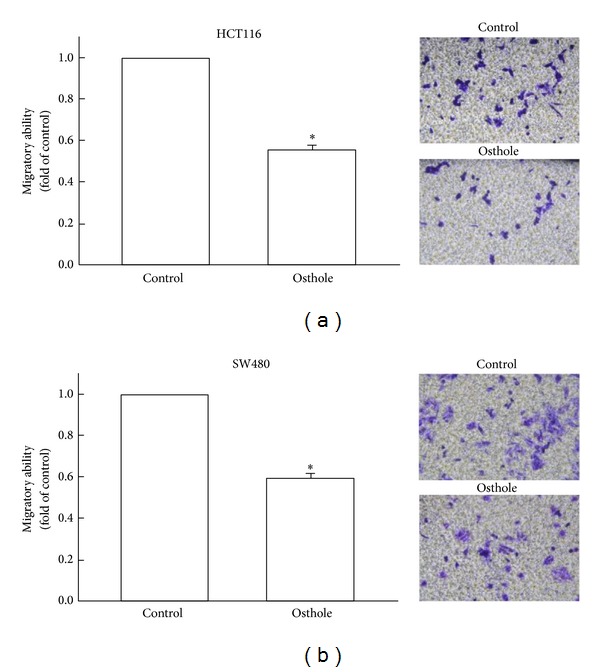
Osthole inhibits migration activity of human colon cancer. By using cell culture insert system, migratory activities of human colon cancer cells were examined. After incubating cells with osthole (10 *μ*M) or vehicle control for 24 h, we found that osthole inhibited migration activity in both HCT116 (a) and SW480 (b) cells. Migrated cells were visualized by phase-contrast imaging (right panels). Results are expressed as means ± S.E.M. from three independent experiments. *P < 0.05, compared with control group.

Osthole-regulated human colon cancer migration was examined by using cell culture insert system. As shown in Figures 2(a) and 2(b), human colon cancer cells (HCT116 and SW480 cells) migrated from the upper to the lower chamber, and images of migrated cells were shown in right panels. Our results indicated that osthole effectively reduced human colon cancer migration in a concentration-dependent manner.

### 3.3. Osthole Induces p53 Protein Activation in Human Colon Cancer

**Figure 3:**
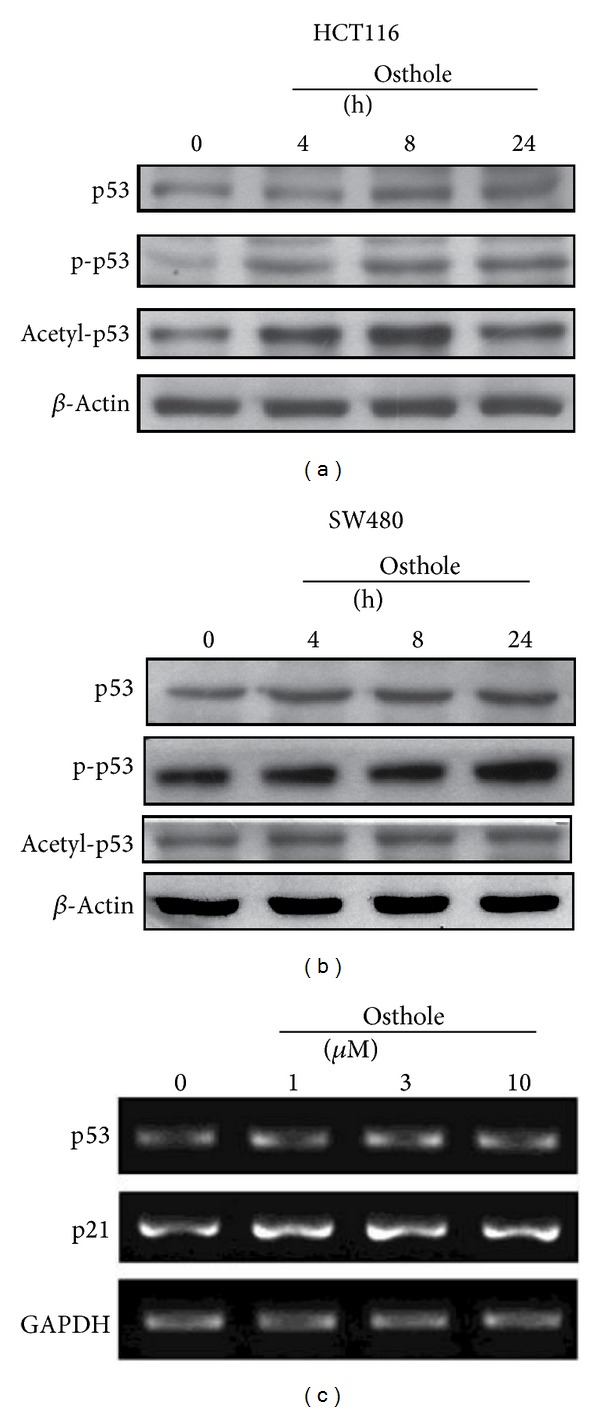
Osthole increases p53 protein activation in human colon cancer. HCT116 (a) and SW480 (b) were incubated with osthole (10 *μ*M) for indicated time periods. p53, phosphorylated p53 (p-p53), and acetylated p53 (acetyl-p53) expressions were examined by western blot analysis. (c) SW480 cells were stimulated by osthole for 6 h, and p53 and p21 expressions were examined by RT-PCR analysis. Results are the representative of three independent experiments.

As shown in Figures 3(a) and 3(b), HCT116 and SW480 cells were incubated with osthole before cell lysate extracts were collected. p53 protein levels, phosphorylation of p53 on Ser15 (p-p53), and acetylation of p53 on Lys^379^ (acetyl-p53) were all increased after osthole stimulation. The effects of osthole on gene expression of p53 and p21 were estimated in SW480 cells using RT-PCR analysis. As shown in Figure 3(c), p53 and p21 gene expression levels were mildly increased in osthole-treated group.

### 3.4. JNK Activation in Osthole Involves Protein p53-Induced Activation

**Figure 4:**
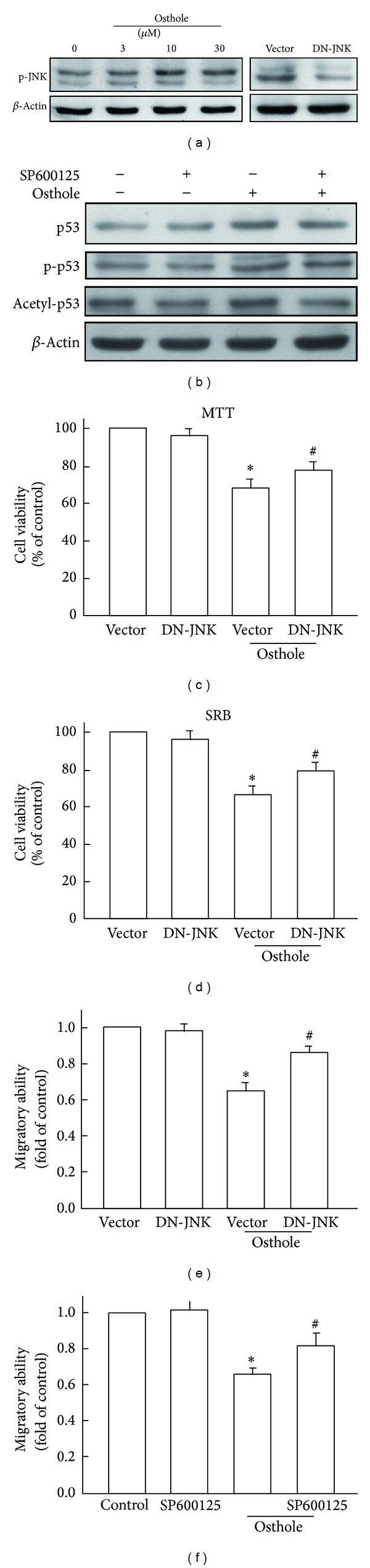
JNK activation contributes to osthole-induced p53 activation in SW480 human colon cancer. (a) Cells were incubated with various concentrations of osthole for 24 h (left panel). Cells were transfected with DN-JNK or empty vector for 24 h (right panel) before cell lysates were collected. Levels of phosphorylated JNK were determined by western blot. (b) Cells were pretreated with or without SP600125 (10 *μ*M) followed by stimulation with osthole for another 24 h; the protein expressions of p53, p-p53, and acetyl-p53 were examined by western blot analysis. Results are the representative of three independent experiments. (c) Cells were transfected with DN-JNK for 24 h followed by stimulation with osthole for another 24 h, and cell viability was examined by MTT (c) or SRB (d) assays. Osthole-inhibited migratory activities were examined by cell culture insert system after transfecting cells with DN-JNK or empty vector for 24 h (e). (f) Cells were pretreated with SP600125 (10 *μ*M) followed by stimulation with osthole for another 24 h, and migratory activities were also examined.

After incubating the SW480 cells with various concentrations of osthole (1, 3, or 10 *μ*M) for 24 h, we found that osthole increased the level of phosphorylated JNK (Figure 4(a)). Moreover, transfection with DN-JNK for 24 h reduced JNK phosphorylation (Figure 4(a)). Treatment with JNK inhibitor SP600125 reduced osthole-induced p53 protein activation (Figure 4(b)). Furthermore, transfection with DN-JNK also attenuated the effects of osthole-induced human colon cancer cell death (Figures 4(c) and 4(d)) and cell migratory effect (Figure 4(e)). Incubation with JNK inhibitor SP600125 also reversed osthole-inhibited cell migratory effect in SW480 human colon cancer cells (Figure 4(f)).

### 3.5. Osthole Induces p53 Protein Activation through Reactive Oxygen Species Production in SW480 Human Colon Cancer

**Figure 5:**
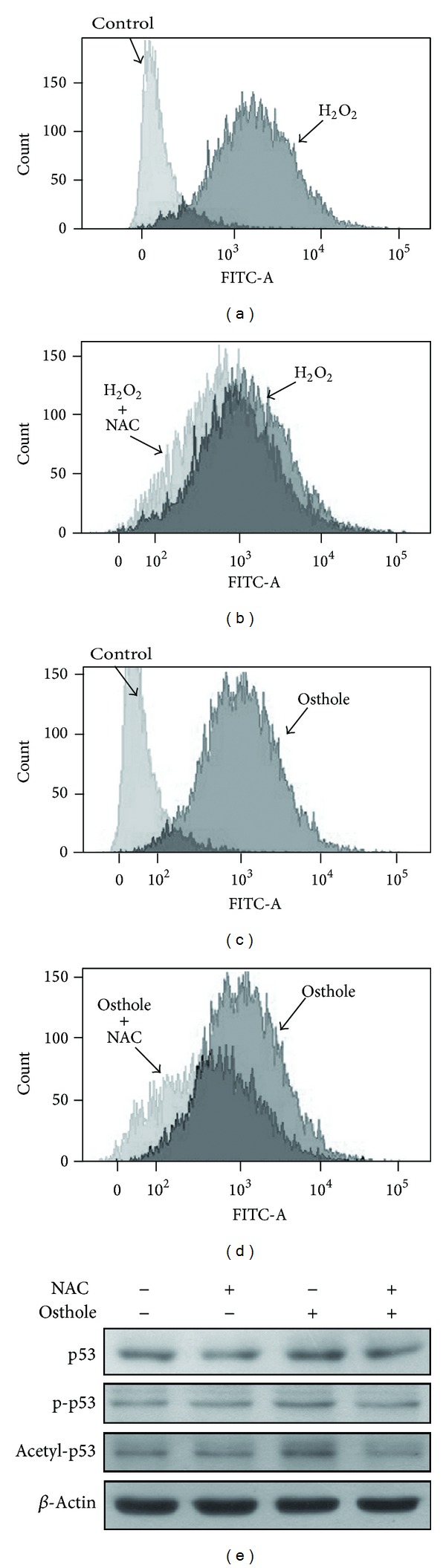
Reactive oxygen species involved in osthole-induced p53 activation. SW480 cells were incubated with H_2_O_2_ (200 *μ*M; (a)), H_2_O_2 _plus N-acetyl-L-cysteine (H_2_O_2_ + NAC; (b)), osthole (10 *μ*M; (c)), or osthole plus N-acetyl-L-cysteine (osthole + NAC; (d)) for 120 min. ROS generations were determined using flow cytometry with the fluorescence probe H_2_DCFH-DA. (e) Cells were treated with NAC plus osthole for 24 h and the protein expressions of p53, p-p53, and acetyl-p53 were examined by western blot analysis. Results are the representative of three independent experiments.

As determined by probe H_2_DCF-DA which was analyzed by flow cytometry assay, osthole was found to increase intracellular ROS levels. Treatment with an ROS scavenger NAC (n-acetylcysteine) reduced hydrogen peroxide (H_2_O_2_) induced ROS production (Figures 5(a) and 5(b)). Moreover, incubation with osthole also dramatically increased ROS generation in human colon cancer cells (Figure 5(c)). Additionally, treatment with NAC antagonized osthole-induced ROS production in SW480 cells as well (Figure 5(d)). As shown in Figure 5(e), treatment with NAC along with osthole in human colon cancer also reduced osthole-enhanced p53 protein expression, p-p53, and acetyl-p53 activation.

## 4. Discussion

Several clinical uses of drugs were derived or modified from plant extracts and have been successfully applied to treat a variety of human cancers [36]. We have reported some natural products and chemical compounds exerting anticancer effects in human glioblastoma [27, 28] and colon cancer [25]. Recently, we also reported that osthole induces cell death and attenuates cell migration in brain tumor [37]. However, the detailed mechanisms underlying the anticancer effects of osthole remain unclear. Our present study reported that osthole increased phosphorylation of p53 on Ser^15^ (p-p53) and acetylation of p53 on Lys^379^ (acetyl-p53), which were regulated by ROS generation and JNK activation. To our knowledge, this is the first report that shows the role of p53 in osthole-induced anticancer effects and identifies the molecular mechanisms through which ROS and JNK modulate p53 protein activation in human colon cancer cells.

Targeting p53, a tumor suppressor, is one of the promising strategies for anticancer therapy; therefore several compounds targeting p53 are currently being tested in clinical studies [38]. Numerous reports have supported this idea of pharmacological restoration of p53 activity for anticancer [39, 40]. Induction of p53 activation leads to cell growth arrest or cell death, but investigation of the detailed molecular mechanism regulating the cancer pathogenesis by p53 remains a key challenge in p53 biology of cancer cells [41]. Importantly, p53-mediated cell fate decisions also prevent cancer cells to be killed by chemotherapeutic drugs, thus leading to poor clinical outcomes. It is imperative to understand the mechanism of p53-mediated cell fate decisions for the efficient clinical application of drugs activating p53 [42]. Our current study reported the crucial role of ROS and JNK in p53-related proapoptotic function in human colon cancer through activation of p53 by a natural product, osthole. Furthermore, our results also demonstrated that JNK-mediated mechanism played a key role in cell death and migratory ability. Interestingly, osthole induced significant cell death and inhibited migratory ability even in the p53-mutated colon cancer cell line SW480 cells.

Several lines of evidence demonstrate that the accumulation of ROS is correlated with the apoptotic response induced by several chemotherapeutic drugs [43]. ROS generation appears to be triggered by the activation of the mitochondrial-dependent cell death pathway through the proapoptotic Bcl-2 proteins Bax and is further transformed sequentially into more toxic ROS, like hydrogen peroxide, which, consequently, induces cell death [44]. Our study showed that osthole elevated the intracellular ROS levels in human colon cancer cells. Furthermore, we observed that blocking ROS production with antioxidant NAC resulted in decreased intracellular ROS levels as well as osthole-induced p53 protein activation. These results indicated that ROS accumulation contributed to osthole-induced cell apoptosis in human colon cancer. Our results also reported that osthole-enhanced proapoptotic protein Bax expression and Bcl-2 degradation. Furthermore, treatment with osthole also increased caspase-3 activation and promoted PARP-1 cleavage. In conclusion, osthole-induced human colon cancer cell death may mediate by ROS generation, which subsequently induces Bax/Bcl-2 turnover and promotes caspase-3 activation and PARP-1 cleavage, resulting in cell apoptosis. Moreover, our results in colon cancer are also in accordance with previous report that osthole effectively attenuates migratory ability in human cancer cells.

## 5. Conclusions 

In the present study, osthole-induced p53 activation results from ROS generation and JNK activation, revealing a promising potential in the treatment of human colon cancer. The current study on a molecular basis provides valuable knowledge of osthole in effective antitumor therapy.

## References

[1] Friend S (1994). p53: a glimpse at the puppet behind the shadow play. *Science*.

[2] Levine AJ (1997). p53, the cellular gatekeeper for growth and division. *Cell*.

[3] Knappskog S, Lønning PE (2012). P53 and its molecular basis to chemoresistance in breast cancer. *Expert Opinion on Therapeutic Targets*.

[4] Livingstone LR, White A, Sprouse J, Livanos E, Jacks T, Tisty TD (1992). Altered cell cycle arrest and gene amplification potential accompany loss of wild-type p53. *Cell*.

[5] Ko LJ, Prives C (1996). p53: puzzle and paradigm. *Genes and Development*.

[6] Garrington TP, Johnson GL (1999). Organization and regulation of mitogen-activated protein kinase signaling pathways. *Current Opinion in Cell Biology*.

[7] Mamay CL, Mingo-Sion AM, Wolf DM, Molina MD, Van Den Berg CL (2003). An inhibitory function for JNK in the regulation of IGF-I signaling in breast cancer. *Oncogene*.

[8] Liu H, Liang S-L, Kumar S, Weyman CM, Liu W, Zhou A (2009). Statins induce apoptosis in ovarian cancer cells through activation of JNK and enhancement of Bim expression. *Cancer Chemotherapy and Pharmacology*.

[9] Gupta S, Campbell D, Derijard B, Davis RJ (1995). Transcription factor ATF2 regulation by the JNK signal transduction pathway. *Science*.

[10] Ham Y-M, Choi J-S, Chun K-H, Joo S-H, Lee S-K (2003). The c-Jun N-terminal kinase 1 activity is differentially regulated by specific mechanisms during apoptosis. *Journal of Biological Chemistry*.

[11] Dinicola S, Pasqualato A, Cucina A (2014). Grape seed extract suppresses MDA-MB231 breast cancer cell migration and invasion. *European Journal of Nutrition*.

[12] Yin M-C (2013). Development of natural antitumor agents. *BioMedicine*.

[13] Sadraei H, Shokoohinia Y, Sajjadi SE, Mozafari M (2013). Antispasmodic effects of Prangos ferulacea acetone extract and its main component osthole on ileum contraction. *Research in Pharmaceutical Sciences*.

[14] Liu J, Zhang W, Zhou L, Wang X, Lian Q (2005). Anti-inflammatory effect and mechanism of osthole in rats. *Zhong Yao Cai*.

[15] Zhang Q, Qin L, He W (2007). Coumarins from Cnidium monnieri and their antiosteoporotic activity. *Planta Medica*.

[16] Luszczki JJ, Andres-Mach M, Cisowski W, Mazol I, Glowniak K, Czuczwar SJ (2009). Osthole suppresses seizures in the mouse maximal electroshock seizure model. *European Journal of Pharmacology*.

[17] Xu X, Zhang Y, Qu D, Jiang T, Li S (2011). Osthole induces G2/M arrest and apoptosis in lung cancer A549 cells by modulating PI3K/Akt pathway. *Journal of Experimental and Clinical Cancer Research*.

[18] Riviere C, Goossens L, Pommery N, Fourneau C, Delelis A, Henichart JP (2006). Antiproliferative effects of isopentenylated coumarins isolated from Phellolophium madagascariense Baker. *Natural Product Research*.

[19] Chou S-Y, Hsu C-S, Wang K-T, Wang M-C, Wang C-C (2007). Antitumor effects of osthol from Cnidium monnieri: an in vitro and in vivo study. *Phytotherapy Research*.

[20] Kamata H, Hirata H (1999). Redox regulation of cellular signalling. *Cellular Signalling*.

[21] Annunziato L, Amoroso S, Pannaccione A (2003). Apoptosis induced in neuronal cells by oxidative stress: role played by caspases and intracellular calcium ions. *Toxicology Letters*.

[22] Monks TJ, Xie R, Tikoo K, Lau SS (2006). Ros-induced histone modifications and their role in cell survival and cell death. *Drug Metabolism Reviews*.

[23] Mo LQ, Chen Y, Song L (2014). Osthole prevents intestinal ischemia-reperfusion-induced lung injury in a rodent model. *Journal of Surgical Research*.

[24] Zhou Y-F, Li L, Feng F (2013). Osthole attenuates spinal cord ischemia-reperfusion injury through mitochondrial biogenesis-independent inhibition of mitochondrial dysfunction in rats. *Journal of Surgical Research*.

[25] Huang S-M, Cheung C-W, Chang C-S (2011). Phloroglucinol derivative MCPP induces cell apoptosis in human colon cancer. *Journal of Cellular Biochemistry*.

[26] Huang SM, Chen TS, Chiu CM (2013). GDNF increases cell motility in human colon cancer through VEGF-VEGFR1 interaction. *Endocrine-Related Cancer*.

[27] Lu D-Y, Chang C-S, Yeh W-L (2012). The novel phloroglucinol derivative BFP induces apoptosis of glioma cancer through reactive oxygen species and endoplasmic reticulum stress pathways. *Phytomedicine*.

[28] Tsai C-F, Yeh W-L, Huang SM, Tan T-W, Lu D-Y (2012). Wogonin induces reactive oxygen species production and cell apoptosis in human Glioma cancer cells. *International Journal of Molecular Sciences*.

[29] Chen J-H, Huang S-M, Tan T-W (2012). Berberine induces heme oxygenase-1 up-regulation through phosphatidylinositol 3-kinase/AKT and NF-E2-related factor-2 signaling pathway in astrocytes. *International Immunopharmacology*.

[30] Lu D-Y, Yeh W-L, Huang S-M, Tang C-H, Lin H-Y, Chou S-J (2012). Osteopontin increases heme oxygenase-1 expression and subsequently induces cell migration and invasion in glioma cells. *Neuro-Oncology*.

[31] Huang BR, Chang PC, Yeh WL (2014). Anti-neuroinflammatory effects of the calcium channel blocker nicardipine on microglial cells: implications for neuroprotection. *PLoS ONE*.

[32] Lu D-Y, Huang B-R, Yeh W-L (2013). Anti-neuroinflammatory effect of a novel caffeamide derivative, KS370G, in microglial cells. *Molecular Neurobiology*.

[33] Lu D-Y, Chen J-H, Tan T-W, Huang C-Y, Yeh W-L, Hsu H-C (2013). Resistin protects against 6-hydroxydopamine-induced cell death in dopaminergic-like MES23.5 cells. *Journal of Cellular Physiology*.

[34] Leung YM, Wong KL, Chen SW (2013). Down-regulation of voltage-gated Ca^2+^ channels in Ca^2+^ store-depleted rat insulinoma RINm5F cells. *BioMedicine*.

[35] Lin H-Y, Yeh W-L, Huang B-R (2012). Desipramine protects neuronal cell death and induces heme oxygenase-1 expression in Mes23.5 dopaminergic neurons. *PLoS ONE*.

[36] Saloustros E, Mavroudis D, Georgoulias V (2008). Paclitaxel and docetaxel in the treatment of breast cancer. *Expert Opinion on Pharmacotherapy*.

[37] Tsai CF, Yeh WL, Chen JH, Lin C, Huang SS, Lu DY (2014). Osthole suppresses the migratory ability of human glioblastoma multiforme cells via inhibition of focal adhesion kinase-mediated matrix metalloproteinase-13 expression. *International Journal of Molecular Sciences*.

[38] Selivanova G (2010). Therapeutic targeting of p53 by small molecules. *Seminars in Cancer Biology*.

[39] Junttila MR, Karnezis AN, Garcia D (2010). Selective activation of p53-mediated tumour suppression in high-grade tumours. *Nature*.

[40] Feldser DM, Kostova KK, Winslow MM (2010). Stage-specific sensitivity to p53 restoration during lung cancer progression. *Nature*.

[41] Vousden KH, Prives C (2009). Blinded by the light: the growing complexity of p53. *Cell*.

[42] Jackson JG, Pant V, Li Q (2012). P53-mediated senescence impairs the apoptotic response to chemotherapy and clinical outcome in breast cancer. *Cancer Cell*.

[43] Fukumura H, Sato M, Kezuka K (2012). Effect of ascorbic acid on reactive oxygen species production in chemotherapy and hyperthermia in prostate cancer cells. *Journal of Physiological Sciences*.

[44] Roos WP, Kaina B (2006). DNA damage-induced cell death by apoptosis. *Trends in Molecular Medicine*.

